# Impact of early term and late preterm birth on infants’ neurodevelopment: evidence from a cohort study in Wuhan, China

**DOI:** 10.1186/s12887-022-03312-3

**Published:** 2022-05-05

**Authors:** Zhong Chen, Chao Xiong, Hua Liu, Junyu Duan, Chun Kang, Cong Yao, Kai Chen, Yawen Chen, Yan Liu, Mingzhu Liu, Aifen Zhou

**Affiliations:** Wuhan Children’s Hospital (Wuhan Maternal and Child Healthcare Hospital), Tongji Medical College, Huazhong University of Science and Technology, No.100, Hong Kong Road, Jiang’an District, Wuhan, 430016 China

**Keywords:** Early term birth, Late preterm birth, Neurodevelopment, Cohort study

## Abstract

**Background:**

The incidences of early term and late preterm birth have increased worldwide during recent years. However, there is a lack of prospective study about the influence of early term and late preterm birth on infants’ neurodevelopment, especially at the early stage. Therefore, we conducted this cohort study to investigate the impact of early term and late preterm birth on infants’ neurodevelopment within 6 months.

**Methods:**

This cohort study was conducted in Wuhan, China, between October 2012 and September 2013. A total of 4243 singleton infants born within 34-41 weeks of gestation at Wuhan Children’s Hospital were included. The Gesell Developmental Scale (GDS) was utilized to evaluate the neurodevelopment of infants.

**Results:**

Among the 4243 included participants, 155 (3.65%) were late preterm infants, 1288 (30.36%) were early term infants, and 2800 (65.99%) were full term infants. After adjusted for potential confounders, significant negative relationship was shown between late preterm birth and development quotient (DQ) in all domains of neurodevelopment: gross motor (*β* = − 17.42, 95% *CI*: − 21.15 to − 13.69), fine motor (*β* = − 23.61, 95% *CI*: − 28.52 to − 18.69), adaptability (*β* = − 10.10, 95% *CI*: − 13.82 to − 6.38), language (*β* = − 6.28, 95% *CI*: − 9.82 to − 2.74) and social behavior (*β* = − 5.99, 95% *CI*: − 9.59 to − 2.39). There was a significant negative trend for early term birth in DQ of fine motor (*β* = − 2.01, 95% *CI*: − 3.93 to − 0.09). Late preterm infants had a significantly elevated risk of neurodevelopmental delay in domains of gross motor (adjusted *OR* = 3.82, 95% *CI*: 2.67 to 5.46), fine motor (adjusted *OR* = 3.51, 95% *CI*: 2.47 to 5.01), and adaptability (adjusted *OR* = 1.60, 95% *CI*: 1.12 to 2.29), whereas early term birth was significantly associated with neurodevelopmental delay of fine motor (adjusted *OR* = 1.22, 95% *CI*: 1.05 to 1.42).

**Conclusions:**

This study suggested that late preterm birth mainly elevated the risk of neurodevelopmental delay of gross motor, fine motor, and adaptability, whereas early term birth was associated with the developmental delay of fine motor within 6 months. Further research is needed to determine the effectiveness and necessity of the interventions at the early stage for early term and late preterm infants who had suspected neurodevelopmental delay.

## Introduction

Preterm birth (less than 37 weeks of gestation) is generally recognized as a major cause of neonatal morbidity and mortality [1]. However, the conventional 37-week cut-off for preterm and term birth has been considered relatively arbitrary in recent years. A growing number of researchers argue that gestational age over 37 weeks does not mean fetal maturity is enough to avoid morbidity [2]. It is indicated that early term infants (37–38^+ 6^ weeks of gestation) have an increased risk of health problems than full term infants (39–41^+ 6^ weeks of gestation). Risks that have been revealed in previous studies include respiratory morbidity, diabetes, obesity-related disorders, long-term developmental outcomes, and even mortality in infancy, childhood, and young adulthood [3–7]. Late preterm birth (34–36^+ 6^ weeks of gestation) is also under-recognized, while previous studies are mainly focused on health outcomes of extremely preterm infants (less than 28 weeks of gestation) [8]. Growing evidence has indicated that late preterm infants have a higher risk of morbidity and mortality than more mature infants [9, 10].

Notably, early term and late preterm births have been prevalent worldwide in recent years. It is estimated that early term and late preterm births account for 15 to 31% and 3 to 6% of all live-born infants, respectively [11, 12]. Despite the ascending proportion of late preterm and early term births, the prospective studies about their short-term and long-term health outcomes are insufficient, especially their impact on infants’ neurocognitive and motor developments. Although a few studies have investigated the association between late preterm birth and delayed neurodevelopment [13–15], the results are still elusive [10]. Moreover, even fewer studies have investigated the relationship between early term birth and neurodevelopment [16, 17]. In addition, previous studies have mainly been devoted to exploring the long-term impacts of late preterm and early term birth on neurodevelopment [14, 18, 19]. In contrast, few have focused on their impact on neurodevelopment at the early stage of infancy [20], which is the optimum period for interventions, as targeted inventions in the early stages of neurodevelopmental delay will achieve the best benefits.

Given the limited reports and the existing inconclusive results on the impact of early term and late preterm birth on neurodevelopment during early infancy, this study aimed to investigate the effect of early term and late preterm birth on infants’ neurodevelopment within 6 months through a cohort study in Wuhan, China.

## Methods

### Study population

Participants of this study were derived from a prospective birth cohort study conducted in Wuhan, China, which was designed to investigate prenatal and postnatal risk factors related to child health. Live singleton infants born within 34–41^+ 6^ weeks of gestation at Wuhan Children’s Hospital between October 2012 and September 2013, without congenital anomalies, were eligible for inclusion in this study.

Initially, a total of 4439 infants met the eligibility criteria after excluding those who refused to accept this routine neurodevelopmental survey (*n* = 8). In the statistical analysis stage, 4243 infants were incorporated after excluding 196 participants who had incomplete demographic information or health records within 6 months. The Medical Ethics Committee of Wuhan Children’s Hospital (Wuhan Maternal and Child Healthcare Hospital) approved the research protocol, and all the procedures involved in this study were executed following the approved guidelines. Informed consent was obtained from the mothers of infants at their first antenatal examination.

### Gestational age classification

Gestational age (GA) was extracted from the delivery record, calculated by the date of delivery and the last recorded menstrual period, and was ascertained by ultrasound examination. According to the GA, infants were categorized as late preterm birth (34 weeks 0 day to 36 weeks 6 days gestation), early term birth (37 weeks 0 day to 38 weeks 6 days gestation), and full term birth (39 weeks 0 day to 41 weeks 6 days gestation) [2].

### Infant development

In this study, the Gesell Developmental Scale (GDS) was conducted to evaluate the neurodevelopment of the infants. The GDS, developed by Gesell and Amatruda, is widely used for child neurodevelopmental diagnosis and intellectual evaluation worldwide [21]. The Chinese version of GDS modified by the Beijing Mental Development Cooperative Group was verified to have good validity and reliability in the Chinese population [22] and has been extensively used to diagnose neurodevelopmental delay among Chinese infants and children (0–6 years) [20, 23]. The Chinese version of GDS items is classified into five domains: gross motor, fine motor, adaptive, language, and social behavior. In brief, the gross motor includes raising head, rolling over, sitting, crawling, standing and walking; fine motor includes finger control and balance; adaptability contains sensation, perception, imitation, and discriminative performance; language is evaluated by pronunciation, word comprehension, and conversation; social behavior contains reactions to people and acquired information. For example, test items of language assessment within 6 months include burbling, calling loudly, vocalizing to people and objects, turning head when names are called, making “da-da” or “ma-ma” sounds, and imitating sounds, etc.

The score in each domain is expressed by developmental quotient (DQ), calculated as below: children’s estimated developmental age/ chronologic age× 100. According to the Chinese norm, the neurodevelopmental delay of each domain was defined as the DQ score below 85 [24]. Four standardized trained clinicians assessed neurodevelopmental tests of this study at the Developmental Neuropsychology Laboratory within 6 months. The evaluators were blind to the prenatal and perinatal characteristics of the infants.

### Covariables

Information about the number of prenatal examinations, parity, mode of delivery, infant’s gender, birth weight, and neonatal asphyxia situation were derived from the delivery record. The socioeconomic factors such as maternal and paternal education level and infant’s feeding pattern were attained by questionnaire.

### Statistical analysis

Mean and standard deviation or frequency and percentage were used to describe the characteristics of the study participants. For each infant, the DQ of the five domains was calculated, and neurodevelopment of each domain was classified into delay (DQ < 85) and normal (DQ ≥ 85) accordingly. One-way analysis of variance (ANOVA) was conducted to compare the mean of DQ of each domain among different GA groups. The generalized linear model (GLM) was used to analyze the prediction of DQ associated with gestational age with adjustment potential confounding factors. Unconditional logistic regression was utilized to calculate the odds ratios (*OR*s) and 95% confidence intervals (*CI*s) for the associations between GA and neurodevelopmental delay in each domain. In GLM and logistic regression analyses, we adjusted for the potential confounders, including parental education level, number of prenatal examinations, parity, mode of delivery, gender of infants, birth weight, neonatal asphyxia, and feeding pattern. In the further stratified analyses by infant’s gender, we evaluated the potential interactions between gender and GA by supplementing the relevant cross-product terms in the logistic regression models. All statistical analyses in this study were conducted using SAS, version 9.4 (SAS Institute, Inc., Cary, North Carolina), and *P*-value < 0.05 was considered statistically significant.

## Results

The distribution of demographic characteristics of participants in this study is shown in Table 1. Among the 4243 participants, 155 (3.65%) were late preterm, 1288 (30.36%) were early term, and 2800 (65.99%) were full term. Of the infants, 2.88% were low birth weight (< 2500 g), and 5.87% were macrosomia (≥ 4000 g). 56.02% of the infants were boys, and 43.98% were girls. A total of 48 (1.13%) infants had asphyxia at birth. There were significant differences in parental education level, the number of prenatal examinations, parity, mode of delivery, infant’s gender, and birth weight among the three groups of late preterm, early term, and full term infants (*P* < 0.01).

**Table 1:** Distribution of demographic characteristics of participants

Variables	Late Preterm (34–36wks)		Early Term (37–38wks)		Full Term (39–41wks)		Total		*P*
	n/mean	%/SD	n/mean	%/SD	n/mean	%/SD	n/mean	%/SD	
***Mothers’ education***									< 0.01
Below bachelor	113	72.90	847	65.76	1731	61.82	2691	63.42	
Bachelor or above	42	27.10	441	34.24	1069	38.18	1552	36.58	
***Fathers’ education***									< 0.01
Below bachelor	108	69.68	830	64.44	1641	58.61	2579	60.78	
Bachelor or above	47	30.32	458	35.56	1159	41.39	1664	39.22	
***Number of prenatal examinations***	7.86	2.47	9.04	2.34	9.28	2.48	9.15	2.45	< 0.01
***Parity***									< 0.01
1	127	81.94	1126	87.42	2630	93.93	3883	91.52	
≥ 2	28	18.06	162	12.58	170	6.07	360	8.48	
***Mode of delivery***									< 0.01
Vaginal	54	34.84	289	22.44	717	25.61	1060	24.98	
Caesarean	101	65.16	999	77.56	2083	74.39	3183	75.02	
***Gender***									< 0.01
Boy	105	67.74	759	58.93	1513	54.04	2377	56.02	
Girl	50	32.26	529	41.07	1287	45.96	1866	43.98	
***Birth Weight (g)***									< 0.01
< 2500	64	41.29	39	3.03	19	0.68	122	2.88	
2500–3999.9	91	58.71	1213	94.18	2568	91.71	3872	91.26	
≥ 4000	0	0.00	36	2.80	213	7.61	249	5.87	
***Neonatal asphyxia***									
No	149	96.13	1275	98.99	2771	98.96	4195	98.87	< 0.01
Yes	6	3.87	13	1.01	29	1.04	48	1.13	
***Feeding Pattern***									0.09
Breast feeding	86	55.48	784	60.87	1741	62.18	2611	61.54	
Mixed feeding	49	31.61	369	28.65	823	29.39	1241	29.25	
Powder milk	20	12.90	135	10.48	236	8.43	391	9.22	

Table 2 presents late preterm birth and early term birth associations with DQ of different neurodevelopmental domains. The mean DQ of early term birth were 100.87, 105.63, 85.88, 87.63, and 88.13 in domains of gross motor, fine motor, adaptability, language, and social behavior, respectively, which were higher than those of late preterm infants (*P* < 0.01), and lower than full term infants (*P* < 0.01). The GLM analysis indicated that late preterm birth was significantly associated with decreased DQ in all domains: gross motor (*β* = − 17.42, 95% *CI*: − 21.15 to − 13.69), fine motor (*β* = − 23.61, 95% *CI*: − 28.52 to − 18.69), adaptability (*β* = − 10.10, 95% *CI*: − 13.82 to − 6.38), language (*β* = − 6.28, 95% *CI*: − 9.82 to − 2.74) and social behavior (*β* = − 5.99, 95% *CI*: − 9.59 to − 2.39). For early term birth, the statistically significant negative trend was only shown in the DQ of fine motor (*β* = − 2.01, 95% *CI* − 3.93 to − 0.09).

**Table 2:** Associations of late preterm birth and early term birth with DQ of different neurodevelopmental domains

Variables	DQ		P^*^	*β*	95% *CI* for *β*		P^**^
	mean	SD			Lower	Upper	
**Gross motor DQ**							
Late Preterm	81.66	22.48	< 0.01	−17.42	−21.15	−13.69	< 0.01
Early Term	100.87	21.64		−0.25	−1.70	1.21	0.74
Full Term	101.64	21.82		0.00			
**Fine motor DQ**							
Late Preterm	82.54	20.89	< 0.01	−23.61	−28.52	−18.69	< 0.01
Early Term	105.63	28.93		−2.01	−3.93	−0.09	0.04
Full Term	108.14	28.95		0.00			
**Adaptability DQ**							
Late Preterm	75.94	21.55	< 0.01	−10.10	−13.82	−6.38	< 0.01
Early Term	85.88	21.54		−0.41	−1.87	1.04	0.58
Full Term	86.49	21.75		0.00			
**Language DQ**							
Late Preterm	81.21	22.78	< 0.01	−6.28	−9.82	−2.74	< 0.01
Early Term	87.63	20.17		−0.59	−1.98	0.79	0.40
Full Term	88.40	20.73		0.00			
**Social behavior development DQ**							
Late Preterm	81.75	22.56	< 0.01	−5.99	−9.59	−2.39	< 0.01
Early Term	88.13	20.12		−0.89	−2.29	0.52	0.22
Full Term	89.16	21.28		0.00			

P^*^, one-way analysis of variance; P^**^, generalized linear model analysis, adjusted for parental education level, the number of prenatal examinations, parity, mode of delivery, infant’s gender, birth weight, neonatal asphyxia and feeding pattern

Results of neurodevelopmental delay in different domains and the association with late preterm birth/early term birth are presented in Table 3. The neurodevelopmental delay rates among late preterm birth and early term birth infants were 56.77 and 22.67% in the gross motor domain, 56.13 and 29.04% in fine motor domain, 64.52 and 55.75% in adaptability domain, 52.26, and 50.85% in the language domain, 49.03 and 48.84% in social behavior development domain, respectively. They were all higher than the corresponding rates among full term infants. According to the results of the binary logistic regression model adjusted for potential confounders, late preterm birth infants had a significantly elevated risk of neurodevelopmental delay in domains of gross motor (adjusted *OR* = 3.82, 95% *CI*: 2.67 to 5.46), fine motor (adjusted *OR* = 3.51, 95% *CI*: 2.47 to 5.01) and adaptability (adjusted *OR* = 1.60, 95% *CI*: 1.12 to 2.29), whereas early term birth was significantly associated with delayed neurodevelopment in fine motor (adjusted *OR* = 1.22, 95% *CI*: 1.05 to 1.42).

**Table 3:** Associations of late preterm birth and early term birth with neurodevelopmental delay of different domains

Variables	Neurodevelopment				Crude *OR* (95% *CI*)	Adjusted *OR*^a^ (95% *CI*)
	Normal		Delay			
	n	%	n	%		
**Gross motor**						
Late Preterm	67	43.23	88	56.77	4.74 (3.40–6.59)	3.82 (2.67–5.46)
Early Term	996	77.33	292	22.67	1.06 (0.90–1.24)	1.01 (0.86–1.19)
Full Term	2192	78.29	608	21.71	1.00 (reference)	1.00 (reference)
**Fine motor**						
Late Preterm	68	43.87	87	56.13	3.88 (2.80–5.39)	3.51 (2.47–5.01)
Early Term	914	70.96	374	29.04	1.24 (1.07–1.44)	1.22 (1.05–1.42)
Full Term	2106	75.21	694	24.79	1.00 (reference)	1.00 (reference)
**Adaptability**						
Late Preterm	55	35.48	100	64.52	1.55 (1.10–2.17)	1.60 (1.12–2.29)
Early Term	570	44.25	718	55.75	1.07 (0.94–1.22)	1.08 (0.94–1.23)
Full Term	1287	45.96	1513	54.04	1.00 (reference)	1.00 (reference)
**Language**						
Late Preterm	74	47.74	81	52.26	1.10 (0.79–1.52)	1.12 (0.79–1.58)
Early Term	633	49.15	655	50.85	1.04 (0.91–1.18)	1.05 (0.92–1.20)
Full Term	1402	50.07	1398	49.93	1.00 (reference)	1.00 (reference)
**Social behavior development**						
Late Preterm	79	50.97	76	49.03	1.04 (0.75–1.43)	1.04 (0.74–1.47)
Early Term	659	51.16	629	48.84	1.03 (0.90–1.17)	1.05 (0.91–1.20)
Full Term	1451	51.82	1349	48.18	1.00 (reference)	1.00 (reference)

^a^Adjusted for parental education level, number of prenatal examinations, parity, mode of delivery, infant’s gender, birth weight, neonatal asphyxia and feeding pattern

We further explored the associations of late preterm birth and early term birth with neurodevelopment delay of different domains stratified by infants’ gender. As shown in Table 4, late preterm boys had a higher risk of neurodevelopment delay in three domains: gross motor (adjusted *OR* = 4.30, 95% *CI*: 2.75 to 6.71), fine motor (adjusted *OR* = 3.86, 95% *CI*: 2.49 to 5.99), and adaptability (adjusted *OR* = 1.64, 95% *CI*: 1.06 to 2.55); late preterm girls had a higher risk of neurodevelopment delay in 2 domains: gross motor (adjusted *OR* = 2.94, 95% *CI*: 1.59 to 5.44) and fine motor (adjusted *OR* = 2.91, 95% *CI*: 1.59 to 5.34). As for early term infants, the significant association was only found with fine motor development delay among girls (adjusted *OR* = 1.32, 95% *CI*: 1.05 to 1.65). The crude *OR* showed that late preterm birth was significantly associated with language delay among girls (crude *OR* = 1.81, 95% *CI*: 1.00 to 3.28). However, the association was no longer significant after adjusting for the potential confounding factors.

**Table 4:** Associations of late preterm birth and early term birth with neurodevelopment delay of different domains stratified by gender

Variables	Boys		Girls		*P* for heterogeneity
	Crude *OR* (95% *CI*)	Adjusted *OR*^a^ (95% *CI*)	Crude *OR* (95% *CI*)	Adjusted *OR*^a^ (95% *CI*)	
**Gross motor**					
Late Preterm	5.25 (3.49–7.89)	4.30 (2.75–6.71)	3.86 (2.18–6.83)	2.94 (1.59–5.44)	0.41
Early Term	1.02 (0.83–1.26)	0.97 (0.79–1.21)	1.11 (0.88–1.42)	1.07 (0.84–1.36)	
Full Term	1.00 (reference)	1.00 (reference)	1.00 (reference)	1.00 (reference)	
**Fine motor**					
Late Preterm	4.19 (2.80–6.28)	3.86 (2.49–5.99)	3.42 (1.93–6.05)	2.91 (1.59–5.34)	0.96
Early Term	1.16 (0.95–1.42)	1.15 (0.94–1.41)	1.37 (1.10–1.71)	1.32 (1.05–1.65)	
Full Term	1.00 (reference)	1.00 (reference)	1.00 (reference)	1.00 (reference)	
**Adaptability**					
Late Preterm	1.64 (1.08–2.48)	1.64 (1.06–2.55)	1.38 (0.77–2.47)	1.51 (0.82–2.79)	0.89
Early Term	1.05 (0.88–1.25)	1.05 (0.87–1.25)	1.10 (0.90–1.35)	1.12 (0.91–1.37)	
Full Term	1.00 (reference)	1.00 (reference)	1.00 (reference)	1.00 (reference)	
**Language**					
Late Preterm	0.90 (0.60–1.34)	0.93 (0.61–1.42)	1.81 (1.00–3.28)	1.68 (0.90–3.13)	0.05
Early Term	0.98 (0.82–1.16)	0.98 (0.82–1.17)	1.15 (0.94–1.41)	1.15 (0.94–1.42)	
Full Term	1.00 (reference)	1.00 (reference)	1.00 (reference)	1.00 (reference)	
**Social behavior development**					
Late Preterm	1.19 (0.80–1.76)	1.10 (0.72–1.68)	0.81 (0.46–1.43)	0.88 (0.48–1.60)	0.76
Early Term	1.01 (0.85–1.20)	1.01 (0.84–1.20)	1.06 (0.87–1.30)	1.10 (0.89–1.35)	
Full Term	1.00 (reference)	1.00 (reference)	1.00 (reference)	1.00 (reference)	

^a^Adjusted for parental education level, number of prenatal examinations, parity, mode of delivery, infant’s gender, birth weight, neonatal asphyxia and feeding pattern

Table 5 shows the association of the specific gestational week with neurodevelopment delay of different domains. After adjusting for potential confounders, infants born between 34 and 37 weeks of gestation showed a higher risk of gross motor and fine motor development delay, and infants born between 34 and 35 weeks of gestation also had a higher risk of adaptability development delay. No significant association was found for language or social behavior development delay.

**Table 5:** Associations of gestational weeks with neurodevelopment delay of different domains^a^

Variables	Gross motor	Fine motor	Adaptability	Language	Social behavior development
34 wks	9.45 (3.69–24.21)	14.78 (4.98–43.84)	5.03 (1.70–14.86)	1.90 (0.83–4.35)	2.00 (0.87–4.59)
35 wks	3.83 (2.07–7.08)	4.24 (2.29–7.84)	2.92 (1.48–5.78)	1.79 (0.97–3.31)	1.19 (0.65–2.15)
36 wks	3.14 (1.99–4.96)	2.38 (1.51–3.75)	0.95 (0.60–1.49)	0.76 (0.48–1.19)	0.81 (0.51–1.27)
37 wks	1.49 (1.14–1.96)	1.72 (1.33–2.24)	1.28 (0.99–1.65)	1.18 (0.92–1.51)	1.10 (0.86–1.40)
38 wks	0.90 (0.75–1.07)	1.11 (0.94–1.31)	1.03 (0.89–1.19)	1.02 (0.89–1.19)	1.04 (0.89–1.20)
39-41wks	1.00 (reference)	1.00 (reference)	1.00 (reference)	1.00 (reference)	1.00 (reference)

^a^Adjusted for parental education level, number of prenatal examinations, parity, mode of delivery, infant’s gender, birth weight, neonatal asphyxia and feeding pattern

## Discussion

In this study, we explored the impact of early term and late preterm birth on the neurodevelopment of infants within 6 months. Our results indicated that late preterm birth was associated with decreased DQ in all neurological domains, and early term birth was associated with decreased DQ in the fine motor. Moreover, late preterm birth had an elevated risk of neurodevelopmental delay in gross motor, fine motor, and adaptability domains. In contrast, early term birth only showed an increased risk of developmental delay in the fine motor.

According to the Fetal Origins Hypothesis, the fetal intrauterine environment, associated with gestational length, has profound and long-term effects on children’s health and development [25]. Our current study showed that late preterm and early term infants exhibited poorer neurodevelopmental levels within 6 months compared with full term infants, enriching the evidence of the association between gestational length and neurodevelopment among children at the early stage. This result is consistent with previous studies conducted among older children [14, 26, 27]. A prospective longitudinal cohort study in Australia found that compared with term infants, moderate and late preterm children had a higher risk of developmental delay in cognitive, language, and motor domains at the age of 2 years [14]. A cohort study in China also suggested that early term birth was associated with an increased risk of delayed neurodevelopment in the psychomotor domain at 2 years old [17]. Neurodevelopmental delay in late preterm infants may be related to brain morphology and structure [28]. Late preterm infants have been reported to have less brain volume and lower differentiated myelination and neural connectivity than full term infants [29, 30]. It has been suggested that the period between 34^0/7^ and 40^0/7^ gestational weeks is a critical period for immature brain growth, in which cortical and white matter volumes are multiplied by two and five, respectively [31]. While early term birth between 37^0/7^ and 38^0/7^ gestational weeks is included in the critical period, the association between early term birth and neurodevelopmental delay may be also explained [31].

Additionally, the prior study has shown a linear correlation between gestational age and birth fetal brain volume [32], supporting the present findings that longer gestation was associated with improved neurodevelopment at the early stage in infants. In line with these findings, a prospective study investigating 232 full term infants in Germany also found that infants with longer gestation had better cognitive and motor development within 1 year old [26]. Moreover, another study in Chilean also reported that the longer pregnancy was associated with higher mental and motor scores of BSID among term infants at 1 year of age [33]. It is suggested that longer gestation can benefit the infants’ neurodevelopment. Specifically, our results showed that infants born at 37 gestational weeks had a significantly elevated risk of neurodevelopment delay in gross motor and fine motor, compared with full term birth infants. This result supports the arguments of the conventional cut-off of 37 gestational weeks for preterm birth. However, taking gestational age as a continuum representing the risk level connected with adverse outcomes may be more appropriate than taking the term and preterm birth as a dichotomy [34]. More attention should be paid to the consequences of gestational length among term infants.

The present study found that late preterm birth mainly influenced infants’ neurodevelopmental delay of gross motor, fine motor, and adaptability during the early stage. In contrast, early term birth was associated with neurodevelopmental delay of fine motor. These findings may suggest that there may be different potential mechanisms under the impact of gestational age for different neurodevelopmental domains, and different neurodevelopmental modules may have different developmental critical periods. It has been shown that sensory axons are usually myelinated before motor axons, and myelination generally extends from the center to the periphery, from the dorsal to the ventral [35]. It seems that the sensory module may develop before the motor module and the gross motor module before the fine motor module. Our results suggest no significant difference between gestational age and neurodevelopmental delay of language at the early stage, which was inconsistent with results of some previous studies [36]. The inconsistency may be related to the physiological age of infants. Unlike most studies focused on the neurodevelopment of infants at least one-year-old, the present study was devoted to assessing the neurodevelopmental level within 6 months. In fact, to the best of our knowledge, there are a growing number of studies evaluating neurodevelopment in infants around 6 months of age [37–39]. A study had shown that infants with neurodevelopmental delays would have better neurodevelopmental outcomes in future if interventions were given at 6 months of age [40]. This might highlight the importance of early identification and intervention of high-risk infants to promote an improved neurodevelopmental outcome.

Further, we investigated the influence of late preterm birth and early term birth with neurodevelopmental delay stratified by infants’ gender. Late preterm birth was associated with a higher risk of delay in 3 domains (gross motor, fine motor, and adaptability) in boys. In contrast, adaptability developmental delay was not observed among late preterm girls. Moreover, the association of early term birth with fine motor developmental delay was only detected in girls. Prior studies have shown that fetal gender may play an essential role in regulating the influence of prenatal exposure on neurodevelopment [41, 42]. Male fetuses are more sensitive to early exposure to risk factors than female fetuses [43]. Wu et al. found that the association between early term birth and the neurodevelopmental delay was more significant in male infants [17]. The results indicated that the impact and mechanism of gestational age on neurodevelopment at an early stage could be different for boys and girls, which needs to be examined and identified in the future.

The main strength of this study is the relatively large sample size compared with previous studies, which included information collected from pregnancy through 6 months after birth. Moreover, we were able to adjust for some potential confounding factors that may impact infants’ neurodevelopment in the present study. Furthermore, the assessment of infants’ neurodevelopment was under strict quality control, making the results more reliable. However, several limitations of this study need to be concerned. First, infants’ neurodevelopment was only assessed within 6 months in this study. As individual neuropsychological development is a long-term process and can be affected by various biological and psychosocial factors, further follow-up is required to identify the neurodevelopmental status of early term and late preterm birth infants in the future. Second, the participants in this study came from a single center of tertiary hospital, limiting our findings’ generalizability to the broader population. Third, although the revised GDS is widely used for neurodevelopmental assessment and diagnosis in China, it is not commonly used in western countries, which increases the difficulty of comparing the results among different studies worldwide.

## Conclusion

In conclusion, we conducted a prospective cohort study in Wuhan, China to evaluate the impact of early term and late preterm birth on infants’ neurodevelopment at the early stage, and found the evidence that late preterm birth mainly elevated the risk of neurodevelopmental delay of gross motor, fine motor, and adaptability. In contrast, early term birth was associated with an increased risk of fine motor developmental delay within 6 months. We found that longer gestation benefits the infants’ gross and fine motor development at an early stage even among term infants. Further research is needed to determine the effectiveness and necessity of the interventions at the early stage for early term and late preterm infants who had suspected neurodevelopmental delay.

## Data Availability

The datasets used and/or analysed during the current study are available from the corresponding author on reasonable request.

## Abbreviations

*OR*: Odds ratio
*CI*: Confidence interval
GDS: Gesell Developmental Scale
DQ: Development quotient
GA: Gestational age
ANOVA: One-way analysis of variance
GLM: Generalized linear model
